# Hyponatremia in Chronic Liver Disease among Patients Presenting to a Tertiary Care Hospital: A Descriptive Cross-sectional Study

**DOI:** 10.31729/jnma.7152

**Published:** 2021-12-31

**Authors:** Abashesh Bhandari, Ashlesha Chaudhary

**Affiliations:** 1 Department of Internal Medicine, Nepal Medical College and Teaching Hospital, Jorpati, Kathmandu, Nepal; 2 Nepal Medical College and Teaching Hospital, Jorpati, Kathmandu, Nepal

**Keywords:** *hyponatremia*, *liver diseases*, *Nepal*, *sodium*

## Abstract

**Introduction:**

Hyponatremia is a frequent problem in chronic liver disease. To the best of our knowledge, no study of hyponatremia in chronic liver disease has been conducted in our setting. The aim of this study is to study the prevalence of hyponatremia in patients with chronic liver disease attending the outpatient department of a tertiary care hospital.

**Methods:**

This descriptive cross-sectional study was conducted in 114 patients with chronic liver disease attending the out-patient department of a tertiary care hospital in Kathmandu, Nepal between November 2020 and July 2021. Ethical approval was taken from the Institutional Review Committee of Nepal Medical College and Teaching Hospital (Reference number: 028-077/078). Convenience sampling was done. The collected data was entered and analyzed in Microsoft Excel. Calculation of point estimate at 95% confidence interval was done along with frequency and proportion for binary data.

**Results:**

Among the 114 patients with chronic liver disease studied, 47 (41.22%) (32.18-50.25 at 95% Confidence Interval) patients had hyponatremia (<130mmol/L) with mean age of 53.44±7.57years. Thirty (63.8%) patients out of these were males and 17 (36.2%) patients were females.

**Conclusions:**

The prevalence of hyponatremia among patients with chronic liver disease was found to be higher when compared to other similar studies.

## INTRODUCTION

Hyponatremia is common in patients with advanced stages of liver diseases. Patients with chronic liver disease (CLD) may develop hyponatremia due to either hypovolemia or hypervolemia. Hyponatremia in CLD is currently defined with a level of serum sodium less than 130 meq/L.^1^

Studies have shown that the severity of the hyponatremia is related to the severity of the chronic liver disease.^2-4^ However, Nepal's data on the occurrence of hyponatremia in chronic liver disease are scarce.^5^ To the best of our knowledge, no study of hyponatremia in chronic liver disease has been conducted in our setting.

The aim of this study is to study the prevalence of hyponatremia in patients with chronic liver disease attending the outpatient department of a tertiary care hospital.

## METHODS

This is a descriptive cross-sectional study conducted among 114 patients with IBS presenting to the Department of Internal Medicine of a tertiary care hospital between November 2020 and July 2021 . Ethical approval was taken from the Institutional Review Committee of Nepal Medical College and Teaching Hospital (Ref No: 028-077/078).

Patients who had given their consent were included in the study. The patients with Child Turcotte Pugh A score, compensated CLD, acute fulminant hepatitis, non-cirrhotic portal hypertension, chronic kidney disease and those who fail to give consent were excluded.

The sampling method for this study was convenience sampling.

The sample size was calculated by using the formula,

n = Z^2^ × p × q / e^2^

  = (1.96)^2^ × 0.367 × (1-0.367) / (0.09)

  = 110

Where,

n= required sample size
Z= 1.96 at 95% Confidence Interval (CI)
p= prevalence of hyponatremia in patients with chronic liver disease based study on previous^6^
q= 1-p
e= margin of error, 9%

A sample size of 114 patients was taken and detailed history with clinical examination were done. Informed consent from patients who were diagnosed with chronic liver disease was taken. Data regarding demographic variables, and varied presentation were documented. Around 5ml of whole blood, which was free from hemolysis was initially centrifuged at 3000 rates per minute at 10 minutes. The separated serum was analyzed for sodium levels using an automated electrolyte analyzer. Hyponatremia among these patients with chronic liver diseases were defined as a serum sodium level of less than 130meq/L.^4^

The data were entered into Microsoft Excel and descriptive statistics were calculated. Point estimate at 95% confidence interval was calculated along with frequency and proportion for binary data.

## RESULTS

Among the 114 patients with chronic liver disease studied, 47 (41.22%) (32.18-50.25 at 95% Confidence Interval) patients had hyponatremia (≤130mmol/L) with mean age of 53.44±7.57years (Table 1).

**Table 1:** Levels of sodium and Child Pugh class among patients with hyponatremia (n= 47).

	Hyponatremia	Child Pugh Class	
	n (%)	B n (%)	C n (%)
≤125mmol/L	24 (51.06)	2 (8.33)	22 (91.66)
126-130mmol/L	23 (48.93)	518 (21.73)	(78.26)
Total	47 (100)	47 (100)	

The mean serum sodium levels among these patients was 124.85 ± 3.27mmol/L. Thirty (63.8%) patients out of these were males and 17 (36.2%) patients were females. All of these patients had a history of alcohol consumption 47 (100%). On upper gastrointestinal endoscopy of these patients, 45 (95.74%) had varices.

The common clinical features in these patients were edema 47 (100%), ascites 44 (93.6%) and pallor 44 (93.6%) as shown in the table below (Table 2).

**Table 2:** Clinical profile among patients of chronic liver disease with hyponatremia (n= 47).

Clinical Features	n (%)
Icterus	34 (72.3)
Ascites	44 (93.6)
Edema	47 (100)
Upper Gastrointestinal Bleeding	35 (74.5)
Hepatic Encephalopathy	22 (46.8)
History of Hepatitis C	0 (0)
History of Hepatitis B	10 (21.3)
Pallor	44 (93.6)
Hepatorenal Syndrome	22 (46.8)
Spontaneous Bacterial Peritonitis	13 (27.7)

These patients were studied for various laboratory parameters and their mean and standard deviation were expressed (Table 3).

**Table 3:** Laboratory parameters among patients of chronic liver disease with hyponatremia (n= 47).

Laboratory Parameters	Mean±SD
Total Leukocyte Count	12770.42±7078
Hemoglobin levels	8.9±1.31
Platelet count	118535.85±149475
Urea	35.34±16.65
Creatinine	1.75±0.9
Albumin	2.56±0.356
Total Bilirubin	6.24±5.42
Prothrombin Time (PT)	21.21±3.07
Alanine Aminotransferase (ALT)	74.74±44.75
Aspartate Aminotransferase (AST)	127.53±48.86
Alkaline phosphatase (ALP)	207.53±62.74

## DISCUSSION

Hyponatremia in chronic liver disease is a condition characterised by increased renal retention of water relative to sodium because of impairment in the clearance of solute-free water, which is frequent in individuals with cirrhosis and portal hypertension.^1^ Hyponatremia can be caused by a variety of reasons among these patients, the most common of which is increased arginine vasopressin secretion (AVP; also known as antidiuretic hormone, or ADH). AVP is considered to be released in portal hypertension via baroreceptor-mediated nonosmotic stimulation induced by a decrease in effective circulation volume, which is generated by arterial splanchnic vasodilation.^1^ Decreased production of solute-free water along with reduction in the delivery of sodium to the distal tubule secondary to reduction in the glomerular filtration rate as well as an increase in the sodium resorption in the proximal tubule are other factors that could be responsible.

Hyponatremia has been showed to cause increase the risk of mortality among individuals with cirrhosis,^7,8^ and in those patients who are on liver transplantation waiting lists.^9^ Its association with greater frequency and severity of complications of cirrhosis has been demonstrated in multiple studies.^10–4^ Singh JP, et al. in a study conducted among 60 patients with chronic liver disease showed 22 (36.7%) patients had the serum sodium levels <130meq/L, 18 (30%) patients with serum sodium levels between 131-135meq/L and 20 (33.3%) patients with serum sodium levels of >135meq/L. Hepatic encephalopathy was seen in 21 patients with serum sodium levels <130meq/L.^6^ Our study showed higher prevalence of hyponatremia ie. among the 114 patients studied, 47 (41.22%) patients had hyponatremia (<130mmol/L).

Younas A, et al. conducted a study in liver disease patients with hyponatremia, which showed serum sodium levels ranging from 115 to 127meq/L (mean 121.41±5.17meq/L).^15^ Low serum sodium levels was present among these patients in 96 (36.9%) patients which was lower that what our study had showed. Fifty-one (53.12%) patients among these were male and 45 (46.8%) were female. Also, mild, moderate and severe hyponatremia among these was present in 24 (9.2%), 56 (21.5%), and 16 (6.2%) patients respectively. The mean serum sodium levels among the patients of chronic liver disease with hyponatremia studied in our study was 124.85 ± 3.27mmol/L. Comparable to this study, our study showed the prevalence of hyponatremia among the patients with liver disease to be higher among males.

In an international prospective study done in 28 liver units, among 997 patients the prevalence of hyponatremia where serum sodium concentration less than 135, 130, 125, and 120mEq/L in patients with liver disease was shown to be 50, 22, 6, and 1 percent, respectively.^10^ Similarly, a total of 100 patients diagnosed with decompensated liver disease were included in another study by Devadas AD, et al. among which males constituted 80% and the rest were females. Sixty-seven% of the patients were Alcoholics. HBsAg positive status was present in 11 patients, Hep C Positive status in 7 patients and, 15 patients were cryptogenic. In this study, hyponatraemia was seen in 70% of the patients which was higher as compared to our study.^16^

A case control study conducted by Shaikh S, et al. which involved 217 patients with chronic liver disease among whom hyponatremia (serum sodium <130meq/l) was seen in 58 (26.7%) patients and 54 (24.9%) patients had serum sodium levels between 131-135 meq/l while 105 (48.4%) patients had serum sodium levels >135meq/l. Our study showed higher prevalence of hyponatremia among patients with chronic liver disease. Among the 114 patients studied, 47 (41.22%) patients had hyponatremia (<130mmol/L).^17^

A study was carried out among 754 patients in the Manipal College of Medical Sciences and Teaching Hospital, Nepal which was a descriptive cross-sectional study and showed the prevalence of hyponatremia (<130mEq/L) in hospitalized patients with decompensated liver disease to be 176 (23.34%), which was lower as compared to the finding from our study.^5^

Borroni G, et al. conducted a study which showed that out of 191 admissions, hyponatremia was seen in 97 (29.8%).^18^ Kim JH, et al. found the prevalence of dilutional hyponatremia, with the serum sodium levels of ≤135mmol/l, ≤130mmol/l and ≤125mmol/l, to be 20.8%, 14.9% and 12.2% respectively.^11^

A study on 997 patients with chronic liver disease was carried out by Angeli P, et al. which found out that the prevalence of hyponatremia as defined by a serum sodium concentration ≤135mmol/l, ≤130 mmol/l, ≤125mmol/l and ≤120mmol/l was 49.4%, 21.6%, 5.7%, and 1.2%, respectively.^10^ Hyponatremia has been clearly documented as an independent risk factor for mortality and is prevalent in individuals with end-stage liver disease.^19^ Low serum sodium levels have been demonstrated to have a significant negative influence on the quality of life in individuals with chronic liver disease and ascites.^20^ A history of encephalopathy, serum creatinine, and bilirubin are all prognostic markers for the development of overt hepatic encephalopathy, as is hyponatraemia with serum sodium ≤130mEq/L.^21^

Small sample size and sampling bias could be the major limitation in our study. Similarly, various other pathologies that could cause hyponatremia could coexist in these patients which would affect the real picture of hyponatremia among patients with chronic liver disease. As this is a descriptive cross-sectional study, association between the low serum sodium levels and chronic liver disease could not be made. Also, the single center nature of this study limits the generalizability of the findings. Therefore, higher studies are warranted for the finding out the real picture of prevalence, association and causality among Nepalese population.

## CONCLUSIONS

The prevalence of hyponatremia among patients with chronic liver disease was found to be higher when compared to other similar studies. Association of hyponatremia in patients with chronic liver disease needs to be tested using higher study designs to establish and decreases morbidity and mortality. causality in our setting. Hyponatremia in patients with to be tested using higher study designs to establish causality in our setting. Hyponatremia in patients with chronic liver disease is correctable and timely correction could improve their functional status and quality of life and decreases morbidity and mortality.
